# CHD9 upregulates *RUNX2* and has a potential role in skeletal evolution

**DOI:** 10.1186/s12860-020-00270-5

**Published:** 2020-04-15

**Authors:** Axel H. Newton, Andrew J. Pask

**Affiliations:** School of BioSciences, The University of Melbourne, Melbourne, Victoria Australia

**Keywords:** CReMM, CBFA1, Homoplasy, Convergence, DNA binding, Osteogenesis

## Abstract

**Background:**

Changes in gene regulation are widely recognized as an important driver of adaptive phenotypic evolution. However, the specific molecular mechanisms that underpin such changes are still poorly understood. Chromatin state plays an essential role in gene regulation, by influencing the accessibility of coding loci to the transcriptional machinery. Changes in the function of chromatin remodellers are therefore strong candidates to drive changes in gene expression associated with phenotypic adaptation. Here, we identify amino acid homoplasies in the chromatin remodeller CHD9, shared between the extinct marsupial thylacine and eutherian wolf which show remarkable skull convergence. CHD9 is involved in osteogenesis, though its role in the process is still poorly understood. We examine whether CHD9 is able to regulate the expression of osteogenic target genes and examine the function of a key substitution in the CHD9 DNA binding domain.

**Results:**

We examined whether CHD9 was able to upregulate its osteogenic target genes, *RUNX2*, Osteocalcin (OC) and *ALP* in HEK293T cells. We found that overexpression of CHD9 upregulated *RUNX2*, the master regulator of osteoblast cell fate, but not the downstream genes OC or *ALP,* supporting the idea that CHD9 regulates osteogenic progenitors rather than terminal osteoblasts. We also found that the evolutionary substitution in the CHD9 DNA binding domain does not alter protein secondary structure, but was able to drive a small but insignificant increase in RUNX2 activation. Finally, CHD9 was unable to activate an episomal RUNX2 promoter-reporter construct, suggesting that CHD9 requires the full chromatin complement for its function.

**Conclusions:**

We provide new evidence to the role of CHD9 in osteogenic differentiation through its newly observed ability to upregulate the expression of *RUNX2*. Though we were unable to identify significant functional consequences of the evolutionary substitution in HEK293T cells, our study provides important steps forward in the functional investigation of protein homoplasy and its role in developmental processes. Mutations in coding genes may be a mechanism for driving adaptive changes in gene expression, and their validation is essential towards determining the functional consequences of evolutionary homoplasy.

## Background

Selection targets traits in response to specific environmental pressures, though the molecular basis of these evolutionary changes remains unclear. Phenotypic development is driven by suites of genes in complex regulatory networks [1–3]. Mutations targeting cis-regulatory regions of key developmental genes are now thought to be the major drivers behind morphological adaptations [1, 4, 5]. However, mutations affecting transcription factors and other protein-coding genes are also known to play critical roles in evolution, particularly through changes in DNA-binding capacity or protein-protein interactions [6, 7]. Recently, studies have examined genome-wide patterns of positive selection and homoplasy in protein coding genes underlying convergent phenotypic traits in mammals. While some key protein-coding genes implicated in similar phenotypes were recovered, overall adaptive homoplasy was rare [8–10]. Despite this, homoplasy and positive selection has been detected in protein-coding genes with plausible links to the convergent phenotypes examined. However, few studies employ functional analyses to determine their potential contribution to the evolution of phenotypic traits.

The development of complex traits is controlled by the expression of multiple genes in concert [6]. Accordingly, modification of traits is likely driven by small incremental changes to the expression and regulation of this suite of genes [3]. The relationships between gene expression and cis-regulation may be controlled through alterations to epigenetic modifiers (chromatin remodellers), proteins which drive broad changes in gene expression within a specific tissue or cell type [11]. One such mechanism to drive concerted changes in gene expression is through genome-wide alterations in chromatin organization [12, 13]. Chromatin state plays an essential role in gene regulation by altering the accessibility of DNA to transcription factors, repressors, insulators, RNA polymerase and other transcriptional machinery. Mutations in genes that remodel chromatin present strong candidate mechanisms which may drive changes in gene expression underpinning phenotypic adaptation.

CHD (chromodomain helicase DNA-binding) proteins are chromatin remodelling enzymes which reshape the local chromatin architecture to allow access to transcriptional machinery in a tissue-specific context [11, 14]. CHD proteins facilitate various stages of gene expression, including binding and recruiting transcription factors, histone modifications, and transcriptional elongation, termination and processing [15]. CHD proteins are characterized by two tandem chromodomains and a helicase C domain, but also possesses additional functional domains which classify them into three subfamilies (reviewed in [14]). Of the nine CHD members, the least-studied is CHD9. CHD9 is active in mesenchymal stromal stem cells and osteoprogenitor cells, and is suggested to control osteogenic cell fate [16–19]. CHD9 contains a DNA-binding domain which has been shown to associate with A/T rich DNA in the promoters of osteogenic genes such as *BGLAP* (osteocalcin; OC), *ALP* (alkaline phosphatase) and the master osteogenic regulator *RUNX2* (runt-related transcription factor 2) [18–22], though how CHD9 influences the expression of these genes is still unknown. RUNX2 is essential for intramembranous ossification of the facial skeleton [23] and has been implicated as a major driver of craniofacial evolution in mammals, most notably in the Carnivora [24–27].

Interestingly, we identified previously undetected homoplasious amino acids in the *CHD9* orthologs of the canids (a family within the Carnivora) and the extinct marsupial thylacine (where it was also found to be under positive selection) [9]. These distantly-related lineages possess remarkable similarities in their skull morphologies [9, 28], representing one of the best examples of convergent evolution seen in mammals [29, 30]. Therefore, evolutionary substitutions in CHD9 present a plausible mechanism behind adaptive changes in craniofacial morphology through modified function. In this study we determine whether overexpression of CHD9 influences the expression of its known osteogenic targets OC, *ALP* and *RUNX2* in vitro; and, examine whether a homoplasious amino acid substitution in the CHD9 DNA-binding domain is able to drive differential gene expression. While adaptive homoplasy is rare [8–10], its occurrence in genes associated with phenotypic traits evokes questions as to how these mutations effect evolutionary gene function. In this study, we provide new evidence into the functional consequences of protein homoplasy, providing insights to the molecular basis of adaptive evolution.

## Results

### Phylogenetic distribution of homoplasious amino acids

**Fig. 1:**
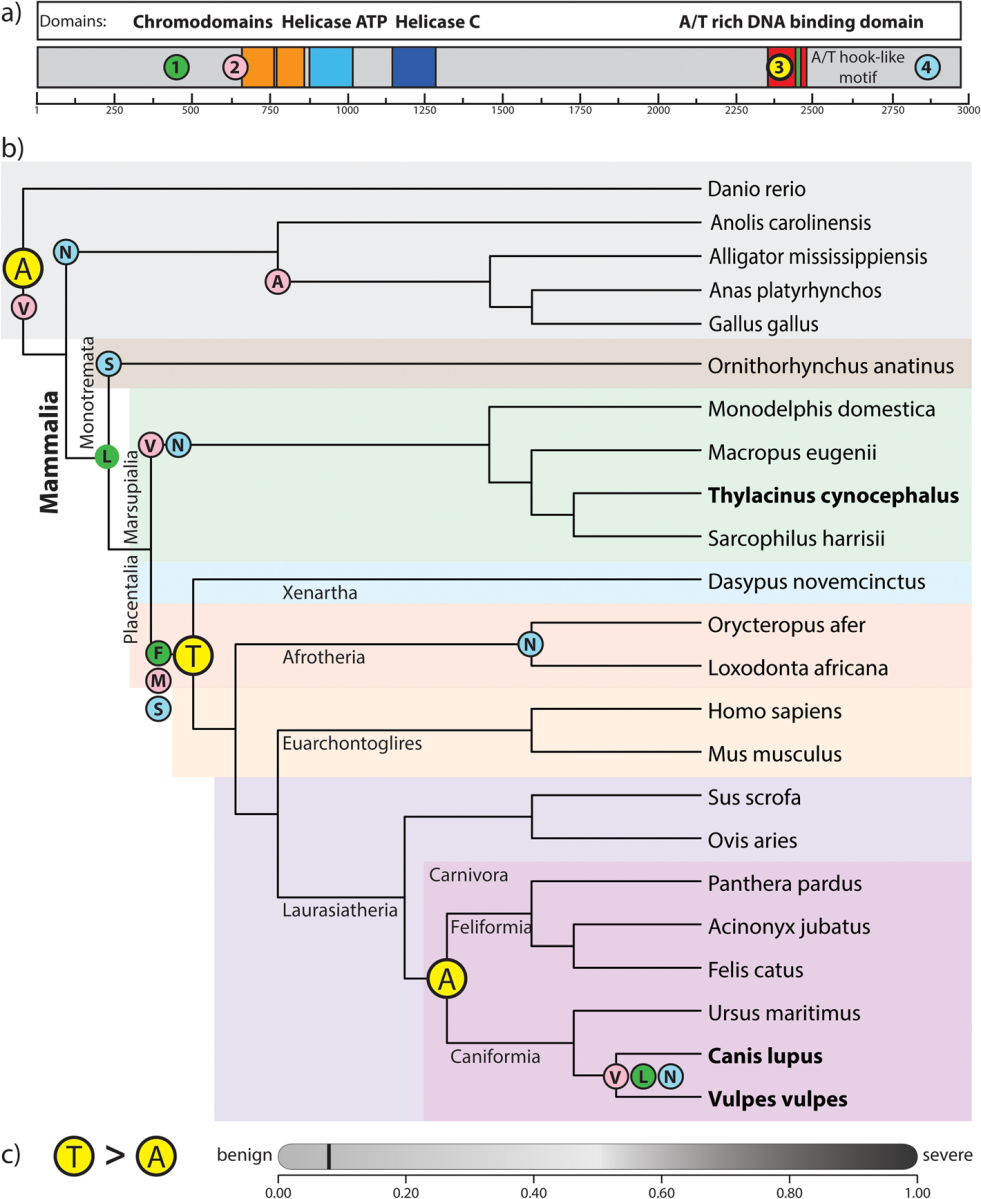
CHD9 protein and simplified mammalian phylogeny showing the four homoplasious amino acid substitutions. **a**) Schematic of the CHD9 protein showing functional domains and location of the four identified homoplasious amino acids. **b**) Simplified mammalian phylogeny showing distribution of each amino acid corresponding to coloured circles in (**a**). Each amino acid shows different distributions throughout the tree and none were specifically shared between the thylacine and canids (bold text). The Ala2384 homoplasious amino acid identified in the DNA-binding domain (3) is the ancestral vertebrate residue and is highlighted in yellow. **c**) The Ala2384 homoplasious substitution was predicted to be a benign mutation (score of 0.053; sensitivity: 0.94; specificity: 0.84), predicted by PolyPhen-2 [31]

We screened alignments of orthologous CHD9 coding sequences to identify shared parallel and convergent amino acid substitutions between the thylacine and canids, with respect to their immediate ancestors. Through this, we identified four individual, homoplasious amino acid substitutions shared between the thylacine and wolf (Fig. 1a). We further examined the evolutionary history of the homoplasious alleles, by investigating their distribution across a simplified vertebrate phylogeny. This revealed that each of the four the amino acid substitutions were not exclusively shared between the thylacine and canids, but rather distributed across additional mammalian lineages (Fig. 1b).

Interestingly, one of the four homoplasious substitutions was found to occur within the DNA-binding domain of CHD9. This substitution at amino acid residue 2384, was found to occur between the thylacine and wolf as a parallel alanine residue (Ala2384). However, additional screening revealed this was in fact the ancestral amino acid among vertebrates. Rather, the last common eutherian ancestor evolved an alanine to threonine substitution (Ala2384Thr), which subsequently reverted back to an alanine residue (Thr2384Ala) in the ancestor of the eutherian Carnivora (including the canids; Fig. 1b). Nevertheless, the location of this amino acid in the DNA binding domain suggests it may possess a functional consequence and provides a tractable candidate to examine the role of evolutionary substitutions.

### The Thr2384Ala substitution does not alter protein secondary structure

Protein coding mutations are often dismissed as major drivers of evolution due to their potential epistatic and pleiotropic effects [6]. As such, we determined the impact of the Thr2384Ala amino acid substitution on protein structure, by running the 141aa CHD9 DNA-binding domain containing the Ala and Thr residue through three protein secondary structure prediction suites, I-TASSER [32], PSIPRED [33] and PolyPhen-2 [31]. Each of these analyses similarly revealed the Thr2384Ala substitution did not alter protein secondary structure and was considered to be a benign change (score of 0.053; sensitivity: 0.94; specificity: 0.84; PolyPhen-2) (Fig. 1c). This is in accordance with the role of coding mutations causing subtle changes to protein structure. Instead, the substitution may influence protein function through altered stability, DNA binding affinity, protein transactivation or protein-protein interactions.

### *Chd9* upregulates *RUNX2* expression

**Fig. 2:**
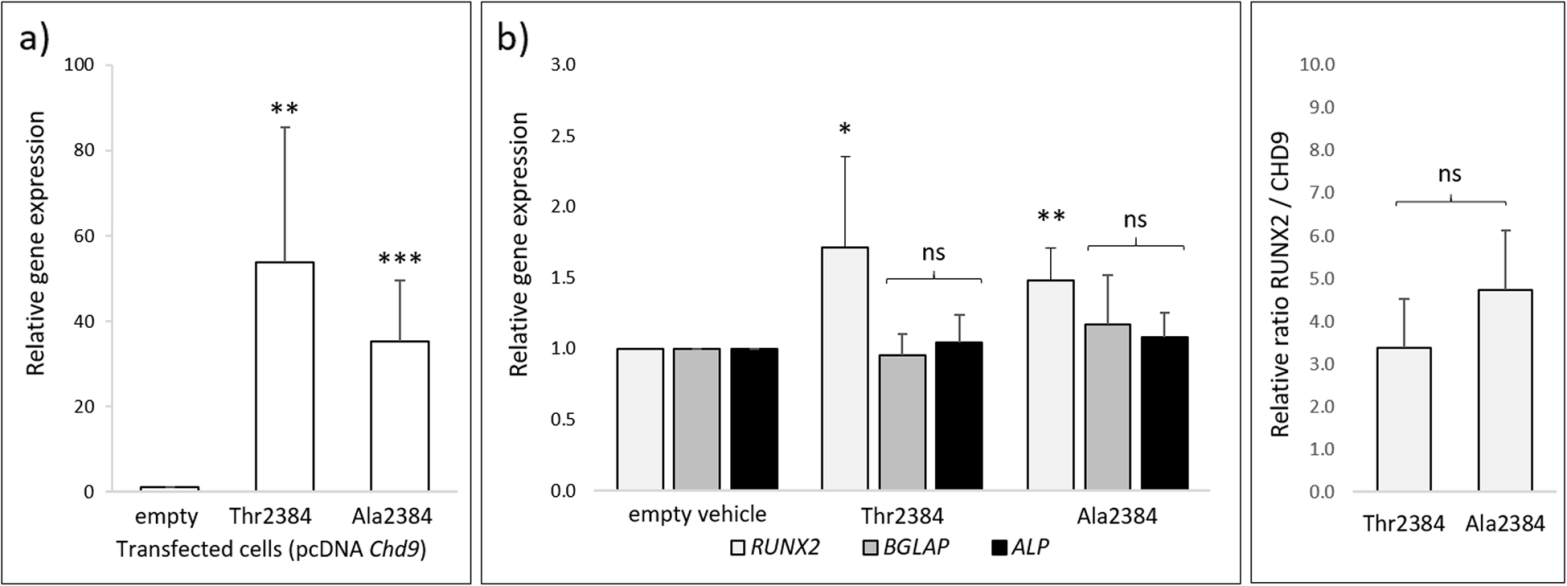
*Chd9* upregulates *RUNX2* expression in HEK293T cells. Expression profiles using ∆∆CT analysis [34] of exogenous *Chd9* and endogenous downstream osteogenic genes, normalized against HPRT gene expression (HK). **a** Exogenous CHD9 expression levels. Exogenous Thr2384 and Ala2384 were strongly over-expressed compared to the empty vehicle, with greater expression in the Thr2384 transfected cells. **b** Endogenous osteogenic gene expression. Expression of Thr2384 and Ala2384 resulted in a significant increase in endogenous human *RUNX2* expression, but not OC or *ALP.***c** Relative *RUNX2* expression*.* RUNX2 was compared against normalized exogenous *Chd9* levels to determine the RUNX2 / CHD9 ratio. The Ala2384 allele resulted in a small but non-significant increase in endogenous *RUNX2* expression compared to the Thr2384 allele

*CHD9* has been shown to associate with the promoter of osteogenic genes [20, 21], but whether it activates their expression was still unknown. To address this, we examined whether exogenous expression of *Chd9* was able to upregulate the endogenous expression of the key osteogenic genes *RUNX2*, OC and *ALP* [19, 21]. To detect any potentially subtle effects, we used the non-osteogenic cell line HEK293T, as CHD9 is not endogenously active. Exogenous overexpression of *Chd9* in HEK293T cells resulted in a ~ 60-fold induction of the Thr2384 allele, and ~ 40 induction of the Ala2384 allele over background *CHD9* levels (Fig. 2a). Interestingly, we found that overexpression of the *Chd9* Thr2384 and Ala2384 allele drove a small but significant ~ 1.5-fold increase in endogenous *RUNX2* expression compared to the empty vehicle control. However, we found that exogenous *Chd9* was unable to upregulate OC and *ALP* in vitro (Fig. 2b).

We next determined whether either CHD9 allele drove differential *RUNX2* expression. To achieve this, we examined the amount of *RUNX2* expression relative to the levels of each *Chd9* allele (Thr2384 or Ala2384) in the transfected cells. While both alleles triggered a significant induction of RUNX2 transcription, the two alleles differed minorly in this capacity. Both induced a ~ 1.5 fold increase, with a minor, though non-significant, increase by the Ala2384 allele (Fig. 2c).

### *Chd9* is unable to transactivate the core *RUNX2* promoter in HEK293T cells

CHD9 was sufficient to upregulate the expression of *RUNX2* in vitro, but its role in the process was still unclear. Specifically, CHD9 functions to remodel chromatin and alter the accessibility of gene-specific promoters to transcriptional machinery, but whether it additionally acts as a transcription factor to promote gene expression is unknown. To test this, we examined whether exogenous *Chd9* was able to directly transactivate the *RUNX2* promoter [35] using an isolated *RUNX2* promoter-reporter assay out of its regular genomic context. We isolated the ~ 780 bp core *RUNX2* promoter for the thylacine (*Thylacinus cynocephalus*) and the red fox (*Vulpes vulpes,* family Canidae*)* and coupled it to a luciferase reporter. We then compared the two *RUNX2* promoter transactivation levels between the Thr2384 and Ala2384 *Chd9* alleles.

**Fig. 3:**
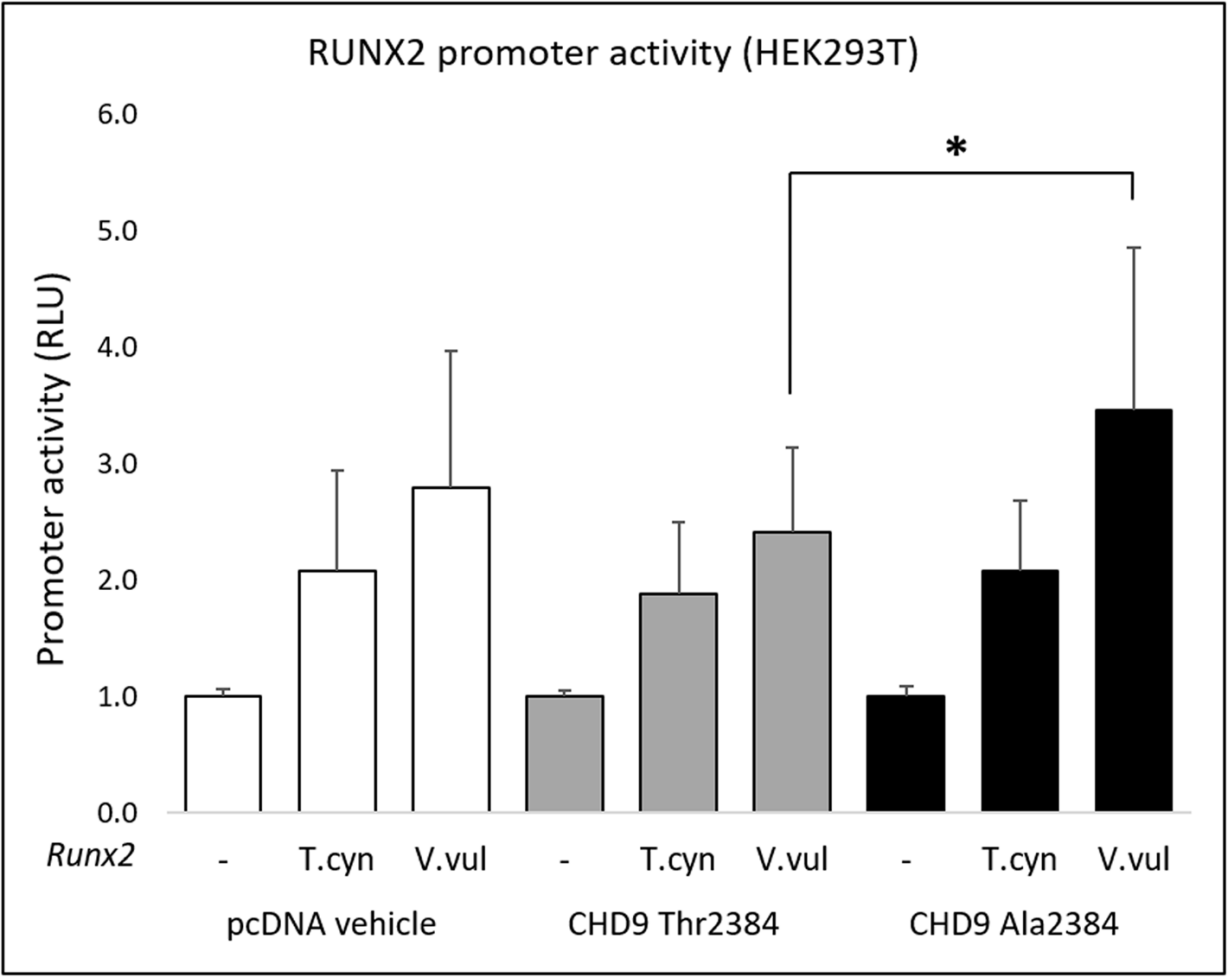
CHD9 does not transactivate the core *RUNX2* promoter in HEK293T cells. Transactivation of the core *RUNX2* promoter by the Ala2384 and Thr2384 *CHD9* variants in HEK293T cells measured by luciferase activity. Exogenous expression of the CHD9 Thr2384 and Ala2384 allele did not cause differential transactivation of the thylacine (*T.cyn*) and red fox (*V.vul) RUNX2* core promoter compared to the empty vehicle control. The Ala2384 allele drove a small but significant increase in red fox *RUNX2* promoter transactivation compared with the Thr2384 allele, though this was not significantly different to the empty vehicle. RLU = relative light units. * denotes significant differences (*P* ≤ 0.05)

The thylacine (*T.cyn*) and red fox (*V.vul*) *RUNX2* promoter-reporter constructs drove low background levels of luciferase activity in HEK293T cells in the absence of exogenous *Chd9* (Fig. 3 left, pcDNA vehicle)*,* with slightly elevated activity in the fox (Fig. 3a)*.* Unexpectedly however, we found that neither the CHD9 Thr2384 or Ala2384 allele were able to drive increased transactivation of the thylacine and fox *RUNX2* promoter, showing similar activation to background pcDNA vehicle (Fig. 3 middle, right). We did detect a small but significant increase in transactivation of the fox RUNX2 promoter by the Ala2384 allele compared to the Thr2384 allele, but this was not significant compared to the background pcDNA vehicle.

## Discussion

Comparative genomic studies have begun to identify the contributions of coding and non-coding mutations to cases of convergent phenotypic evolution in mammals [8, 10, 36–39], though few have functionally validated these potential evolutionary candidates [7]. Without such analyses, the role these mutations play in adaptive evolution remains unclear. In this study we identified and examined the functional consequence of a homoplasious amino acid substitution in the osteogenic chromatin remodeller *CHD9. CHD9* appears to play an important convergent evolutionary role as it has been previously shown to be under positive selection in the thylacine [9], and shares four homoplasious amino acid substitutions with the canids (Fig. 1). Of these substitutions, we identified a key homoplasious alanine residue (Ala2384) in the DNA-binding domain of *CHD9* which was found to be benign and not alter protein secondary structure (Fig. 1c). Rather, this substitution causes a minor modification within a functional domain and may instead influence function through modified protein stability, DNA binding affinity, protein transactivation or protein-protein interactions [6]. As such, the occurrence of this substitution provides a tangible system to examine the functional consequence of evolutionary mutations in vitro.

*CHD9* is an understudied member of the CHD family of chromatin remodellers [11, 14, 15, 20], but has been suggested to remodel and bind to the promoters of osteogenic genes in mesenchymal stem cells and osteoprogenitors [17–21]. However, the precise biological roles CHD9 play remain relatively unknown, including whether it directly promotes transcription of its osteogenic gene targets. We examined the transactivation potential of CHD9 through exogenous expression assays in HEK293T cells. HEK293T cells represent a non-osteogenic cell type, but are robust and routinely used for in vitro gene expression assays. Particularly, the lack of endogenous CHD9 or other osteogenic genes allow for detection of potentially subtle interactions in vitro. Here, we found that CHD9 induced significant upregulation of *RUNX2,* but not the more downstream osteogenic genes OC and *ALP* (Fig. 2b). This result confirms that CHD9 not only remodels chromatin [17, 18], but also functions to regulate some of its osteogenic gene targets [21]. Given CHD9 was only able to upregulate the expression of the early osteogenic regulator *RUNX2*, rather than the downstream osteoblast-specific OC [40] or *ALP*, this suggests that CHD9 is important in driving the early stages of osteogenesis. This is in accordance with findings that CHD9 is active in the differentiation of osteoprogenitors and mesenchymal stem cells, rather than terminal matrix-depositing osteoblasts [16–19].

With the newly observed role of CHD9 in *RUNX2* regulation, we examined whether the homoplasious Ala2384 substitution produces altered *RUNX2* expression. We found both alleles were able to increase *RUNX2* expression, further confirming CHD9 regulates *RUNX2*, with the Ala2384 allele driving a small upregulation compared to the Thr2384 allele, however this was not significant (Fig. 2c). Despite the small increase, the lack of significance limits any definitive conclusions as to the whether the amino acid produces functional consequences. However, this result is consistent with the theory that phenotypic disparity is driven by multiple small and incremental changes that act in concert [1, 2, 6]. Additionally, these subtle changes in *RUNX2* expression may translate to larger effects in vivo*,* or when CHD9 is expressed in an osteogenic context. RUNX2 is critical in development of the skeleton [23] and has been implicated in craniofacial evolution within groups of mammals [24–27, 41]. As such, CHD9-mediated alterations to *RUNX2* expression and regulation may play a role in differential skeletogenesis between species [41]. However, the larger evolutionary role this substitution may play requires further investigation.

In addition to its known association with osteogenic gene-specific promoters and remodelling capabilities [18, 20, 21], we explored whether CHD9 possesses an additional role as a transcription factor, able to directly transactivate *RUNX2* through its core promoter [42]. We found that CHD9 was unable to activate the isolated *RUNX2* promoter (Fig. 3), suggesting this might not be the case. However, the lack of activity could also be explained by the exogenous conditions of the system for a few reasons. Firstly, the ≥750 bp core *RUNX2* promoter is a ‘bone-specific element’ active only in osteoblasts [35]. In contrast, the complete endogenous *RUNX2* promoter has been described as a 3 kb element which contains multiple upstream tissue-specific response elements [35, 43] which may be required to facilitate the *CHD9*-induced activation we observed. Secondly, CHD9 regulatory activity may require an endogenous chromatin environment, since this gene acts as a chromatin remodeller. Thus, it may not be able to function in an extra-chromosomal (episomal) context with a transfected *RUNX2* promoter. CHD9 function has been tightly associated with active histone modifications at promoters [14, 17, 18]. As such, CHD9 may require chromatin-bound transcriptional co-factors to activate its target promoters and gene transcription. Finally, a more biologically relevant role of CHD9 during osteogenesis may be resolved through the use of other in vitro models such as mesenchymal stem cells [20] or osteoprogenitors cells [19] where CHD9 and RUNX2 are both active. Nevertheless, our findings provide new evidence that CHD9 regulates *RUNX2* expression, further implicating it in osteogenesis [16, 20, 21].

## Conclusion

CHD9 is a chromatin remodeller and epigenetic modifier suggested to be active in osteogenesis and skeletal development [16, 19–21, 44], though the precise roles it plays throughout these processes are still poorly understood. Our data provide a novel new role for CHD9 in osteogenesis though its ability to regulate *RUNX2* expression. Additionally, our examinations of CHD9 homoplasy revealed a candidate amino acid substitution in the *CHD9* DNA binding domain prompting functional validation. While we saw a small increase in *RUNX2* activation by the homoplasious allele, this was not significant. Homoplasious amino acid substitutions in coding genes are generally rare and not enriched between convergent traits [8–10], though their occasional presence requires experimental validation. In this study, we take necessary steps investigating the functional roles of protein homoplasy but highlight that these effects may be subtle and difficult to identify. The presence of additional untested homoplasies in CHD9 also evoke interesting questions as to how these substitutions may function in concert. Nevertheless, our newly observed ability for CHD9 to regulate *RUNX2,* combined with its potential to differentially activate its expression, provides an attractive mechanism by which adaptive phenotypic evolution may be controlled at the molecular level.

## Methods

### Identification of gene homoplasy

The method for identifying thylacine-canid amino acid homoplasies has been previously described [9]. Briefly, CHD9 Sequences from the thylacine and canids (wolf, coyote, jackal, red fox and arctic fox) were extracted from previously published reference-based genome assemblies. High-confidence 1:1 orthologous CHD9 sequences from a variety of other mammals (Tasmanian devil, wallaby, opossum, elephant, human, bat, sheep, horse, ferret and dog) were downloaded from Ensembl 84 BioMart. Codon alignments of all sequences were then produced using the translation-aware aligner MACSE version 1.01b with default parameters. Published phylogenies for all included species were assumed [45] and ancestral sequence reconstructions were predicted for all internal nodes using CodeML (PAMLv4.7). Each alignment column was then examined for amino acids shared between the thylacine and canids, but which differed from that of their ancestors or living relatives. Previously, all homoplasious acids were defined as residues shared between the thylacine and canids, but which differed with respect to their immediate ancestors [9]. Through this approach we identified new thylacine and canid protein coding homoplasies to those previously reported [9] due to the strict focus on convergent and parallel amino acid changes. Cases where the ancestral states were the same were defined as parallel, while cases where their ancestral amino acids differed were defined as convergent.

### Phylogenetic distribution and structural properties of the substitution

The use of high confidence 1:1 mammalian orthologs to detect CHD9 homoplasy limited our number of species in our screen. We therefore examined the distribution of the 2384 amino acid residue across a broader vertebrate phylogeny. CHD9 coding sequences from a wide range of vertebrate species were extracted from GenBank and aligned. We then constructed a simplified vertebrate phylogenetic tree using PhyloT (biobyte solutions, GmbH) based on NCBI taxonomy, to visualize the specific amino acid for each linage throughout the branches (Fig. 1b).

To determine whether the homoplasious amino acid altered the physical properties of CHD9, we investigated protein folding and secondary structure through computational tools. The 141 amino acid DNA binding domain containing both the Thr2384 and Ala2384 amino acid motif was input into PSIPRED [33] and I-TASSER [32] to determine secondary structure of the domain. In parallel, we input the full length CHD9 CDS into PolyPhen-2 [31] to predict the impact of the substitution on CHD9 structure and function.

### CHD9 cloning

All functional experiments were performed using the mouse *Chd9* CDS, which natively contains the Thr2384 allele. To generate the *Chd9* coding sequence, *Chd9* mRNA was isolated from MC3T3-E1 cells and converted to cDNA, serving as the amplification template. To obtain the full length *Chd9* CDS, two separate PCR reactions using Phusion HSII High Fidelity polymerase (Invitrogen) were performed on the 5′ (4.5 kb) and 3′ (4.7 kb) ends of the transcript to obtain two 600 bp overlapping amplicons. A second round of PCR was performed using outer primers to obtain the full-length (8.65 kb) product including the Thr2384 residue. Correct sequence identify was confirmed by capillary electrophoresis sequencing (Centre for Translational Pathology, University of Melbourne) using 12 unique tiling primers. The full-length mouse Chd9 CDS was subcloned into the pGEM-T easy vector (Promega) for propagation and purification. For primer sequences see Supplementary Table 1.

We next generated the Ala2384 amino acid allele. To do this we designed and synthesized a 490 bp gBlock (Integrated DNA Technologies, Iowa, USA), containing a Thr > Ala codon substitution at the 2384 residue. The gBlock additionally contained two unique flanking restriction enzyme sites - BbeI (5′) and SfoI (3′) (NEB). The pGEM_*Chd9*Thr2384 plasmid was linearized with BbeI and SfoI to remove the Thr2384 codon and then purified (QIAQuick Gel Extraction kit, QIAGEN). The gBlock containing the Ala2384 residue was digested with BbeI and SfoI and ligated into the linearized pGEM-*Chd9* to obtain the full length CDS with Ala2384 amino acid pGEM_*Chd9*Ala2384. The full length Ala2384 product was sequenced to confirm the presence of the Ala2384 substitution.

The pGEM_*Chd9*Thr2384 and pGEM_*Chd9*Ala2384 plasmids were further digested with *NotI* (NEB) to excise the full-length CDS from the vector backbone. The resulting full-length products were ligated and subcloned into the pcDNA3.1(+) Mammalian Expression Vector (ThermoFisher) for downstream experimental analyses.

### *RUNX2* promoter-reporter construct design

We generated *RUNX2* promoter-reporter constructs from the thylacine (*Thylacinus cynocephalus)* and canid the red fox (*Vulpes vulpes)* [9]*.* The core *RUNX2* promoter has been previously described as a ~ 960 bp element in mouse and ≥ 750 bp element in other mammals [35]. The thylacine promoter was extracted from the thylacine referenced genome assembly using the Tasmanian devil sequence [9]. This was aligned against other placental and marsupial sequences to determine the core marsupial / placental *RUNX2* promoter. We determined the promoter as a ~ 780 bp element in the thylacine and ~ 800 bp element in the red fox, which despite their large evolutionary divergence are highly conserved. The thylacine 780 bp element was synthesized as a single gBlock (Integrated DNA Technologies, Iowa, USA), while the fox element was amplified from red fox gDNA with canid specific primers (Supplementary Table 1). Both products contained flanking *NheI* and *HindIII* restriction sites for subcloning. The thylacine and canid *RUNX2* promoter constructs were digested and ligated into the luciferase reporter pGL4.10[luc2] (Promega).

### Cell culture and transfection

Human embryonic kidney (HEK293T) cells were maintained in DMEM (ThermoFisher) media containing 10% FBS. The media was replaced every 2–3 days. Cells were passaged at 90% confluency. HEK293T cells were seeded in 6 well plates and transfected using lipofectamine 3000 (ThermoFisher) according to the manufacturer’s instructions. Cells were either transfected with 2.5μg of empty vector (pcDNA), or plasmids containing the Thr2384 or Ala2384 alleles and incubated for 24 h. Each transfection experiment was repeated 5 times for reproducibility.

### RNA extraction, gene expression and quantification

Transfected cells were harvested, and RNA was extracted using the GenElute Mammalian Total RNA Miniprep Kit (Sigma Aldrich). RNA was treated with Turbo DNA-free DNase (Ambion) to remove all residual traces of gDNA. The resulting RNA was stored at − 80 °C. Total RNA was converted to cDNA using the SuperScript™ III First-Strand Synthesis System (ThermoFisher). RT-qPCR was performed to examine expression levels for each of the genes of interest in cells transfected with empty vehicle, or *Chd9* possessing the Thr2384 and Ala2384 alleles (*n* = 5). Thr2384 or Ala2384 transfected cell gene expression values were normalized against the housekeeping gene *HPRT* and the un-transfected vehicle using the ΔΔ*CT* method [34]. As exogenous levels of Thr2384 and Ala2384 CHD9 were not equal in our transfection replicates, we additionally compared normalized expression levels of *RUNX2* directly against the normalized exogenous levels of the Thr2384 and Ala2384 CHD9 alleles and determined their levels as a ratio. Primer sequences are listed in Supplementary Table 1.

### Luciferase promoter-reporter assays

HEK293T cells were seeded in white flat-bottom 96-well plates and allowed to recover for 24 h. The following day, cells were transfected using Lipofectamine 3000, according to manufacturer instructions, with either empty pcDNA, Thr2384 or Ala2384; as well as either the empty pGL4.10 vector, thylacine or fox *RUNX2* promoter-luciferase reporter. *RUNX2* promoter driven firefly luciferase and Renilla luciferase activity were analysed 48 h later using the Dual-Luciferase Reporter Assay System (Promega) on a FLUOstar OPTIMA dual plate reader (BMG Labtech Ortenberg, Germany). Firefly luciferase values were normalized against Renilla luciferase to obtain relative promoter activity, measured as relative light units (RLU). Each transfection and luciferase assay were repeated 5 times for reproducibility.

## Supplementary information

**Additional file 1.** Supplementary Table 1 – Primer sequences used in the study.

## Data Availability

All data generated or analysed during this study are included in this published article [and its supplementary information files].

## Abbreviations

CHD9: Chromatin helicase DNA-binding protein 9
RUNX2: Runt-related transcription factor 2
RT: Reverse transcription
PCR: Polymerase chain reaction
