# Supplementary material for: CHD9 upregulates RUNX2 and has a potential role in skeletal evolution
Source: BMC Mol Cell Biol. 2020 Apr 15;21:27. doi: 10.1186/s12860-020-00270-5 (PMC7161146; doi:10.1186/s12860-020-00270-5)
Supplement: Supplementary file 1 — Additional file 1. Supplementary Table 1 – Primer sequences used in the study. [file 12860_2020_270_MOESM1_ESM.pdf]

Primer	F primer (5’ – 3’)	R Primer (5’ – 3’)
CHD9 5’ amplicon	ATGACAGACCCAATGATGGACTTTTTTGAT	CGATGGCGAGAGATCCTGTC
CHD9 3’ amplicon	TTGACCGAGCCAGCTTGAAA	GAGGACTCAGACTCGAGCAATGAAGACTGA
H.s CHD9 qPCR	ACCAAATCTGTTGGGCATGG	GAGTTTTCTCCACCATTCTC
M.m CHD9 qPCR	ATGCGAGAAAGGTTGGAGGC	CACAACGGGACCAGTGAGAA
H.s RUNX2	ATGTGTGTTTGTTTCAGCAGCA	TCCCTAAAGTCACTCGGTATGTGTA
M.m RUNX2	CCCAGCCACCTTTACCTACA	TATGGAGTGCTGCTGGTCTG
H.s BGLAP2	CGCCTGGGTCTCTTCACTAC	CTCACACTCCTCGCCCTATT
M.m BGLAP2	AAGCAGGAGGGCAATAAGGT	TAGGCGGTCTTCAAGCCATA
H.s ALP	ATGGGATGGGTGTCTCCACA	CCACGAAGGGGAACTTGTC
M.m ALP	AACCCAGACACAAGCATTCC	GAGAGCGAAGGGTCAGTCAG
H.s HPRT	TATGGCGACCCGCAGCCCT	CATCTCGAGCAAGACGTTCAG
V.vul RUNX2 promoter	GAGCTCGCTAGCAGATCCTCAAACTGGTCATGAGATAAAC	ATTGGCAAGCTTAGCTTTAAAACTCCTTTTTTTTGCAAGCAC
